# The relationship between airway immunoglobulin activity and eosinophils in COPD

**DOI:** 10.1111/jcmm.16206

**Published:** 2020-12-27

**Authors:** Thomas Southworth, Andrew Higham, Umme Kolsum, Jian Li, Thomas Scott, Josiah Dungwa, Sriram Sridhar, Tuyet‐Hang Pham, Paul Newbold, Dave Singh

**Affiliations:** ^1^ Division of Infection, Immunity and Respiratory Medicine Manchester University NHS Foundation Trust University of Manchester Manchester UK; ^2^ Medicines Evaluation Unit Manchester UK; ^3^ Translational Science Oncology R&D AstraZeneca Gaithersburg MD USA; ^4^ Translational Science & Experimental Medicine Early Respiratory & Immunology Research & Early Development, BioPharmaceuticals R&D AstraZeneca Gaithersburg MD USA; ^5^ BioPharmaceuticals Medical AstraZeneca Gaithersburg MD USA

**Keywords:** B‐lymphocytes, haemophilus influenza, immunoglobulin A, immunoglobulin G, immunoglobulin M

## Abstract

In chronic obstructive pulmonary disease (COPD), the effects of inhaled corticosteroids are predicted by blood eosinophil counts. We previously briefly reported increased immunoglobulin (Ig)A and IgM levels in bronchoalveolar lavage (BAL) of COPD patients with higher (eosinophil^high^) compared to lower (eosinophil^low^) blood eosinophils (>250/μL versus < 150/μL), suggesting differences in adaptive immune function. An inverse relationship exists between eosinophil counts and airway pathogenic bacteria levels. The mechanistic reasons for these associations between eosinophils, corticosteroids and pathogenic bacteria are unclear. IgA, IgM and IgG levels were assessed in BAL, bronchial biopsies and epithelium collected from eosinophil^high^ (*n* = 20) and eosinophil^low^ (*n* = 21) patients. Bronchial B‐cell numbers were measured by immunohistochemistry. B‐cell activity was assessed in bronchial samples and following exposure to BAL from eosinophil^high^ and eosinophil^low^ patients. BAL levels of non‐typeable Haemophilus influenza (NTHi)‐specific immunoglobulins were quantified. Results showed airway expression of IgA, IgG1 and IgM were lower in eosinophil^low^ compared to eosinophil^high^ patients, with lower levels of NTHi‐specific IgA and IgM. Bronchial B‐cell numbers were similar in both groups, but B‐cell activity was lower in eosinophil^low^ patients. In conclusion, COPD eosinophil^low^ patients show differences in adaptive immune function compared to COPD eosinophil^high^ patients. These differences may cause different microbiomes in these COPD phenotypes.

## 1 INTRODUCTION

Randomized controlled trials have shown that blood eosinophil counts are a biomarker that predict the effects of inhaled corticosteroids (ICS) in chronic obstructive pulmonary disease (COPD) patients at increased exacerbation risk.^1^, ^2^ The Global initiative for the management of Obstructive Lung Disease (GOLD) report recommends the use of blood eosinophil measurements to help guide the use of ICS containing combination treatments in COPD patients.^2^

The mechanistic reasons for the increased ICS response in COPD patients with higher blood eosinophil counts are unclear. We previously performed a bronchoscopy study in 21 blood eosinophil low (<150 eosinophils/µl; eosinophil^low^) and 20 blood eosinophil high (>250 eosinophils/µl; eosinophil^high^) COPD patients to investigate biological differences associated with eosinophil counts.^3^ We reported higher lung eosinophil numbers, thicker reticular basement membrane and differences in the levels of various inflammatory mediators including IL‐5, IL‐13, CCL24 and CCL26 in eosinophil^high^ compared to eosinophil^low^ patients.^3^, ^4^ Such findings are also observed in patients with asthma,^5^ providing insights into potential reasons for differential ICS responses associated with blood eosinophil counts in COPD.

Our previous report noted higher IgA and IgM levels in the bronchoalveolar lavage (BAL) of COPD eosinophil^high^ compared to eosinophil^low^ patients,^3^ suggesting differences in adaptive immunity between these groups. An inverse association has been reported between sputum eosinophil counts and the levels of pathogenic bacteria in the airways.^6^, ^7^, ^8^, ^9^ Additionally, COPD patients with blood eosinophil counts <100 cells/µl are more likely to have chronic bacterial airway infection.^10^ Overall, these findings support the concept that COPD patients with lower eosinophil levels are more susceptible to bacterial airway infection. Furthermore, COPD or asthma patients with raised blood and sputum eosinophils demonstrate greater presence of bacterial phylum Bacteroidetes,^11^ whereas low sputum eosinophil levels were associated with lower bacterial diversity and increased Proteobacteria, specifically the Haemophilus genus.^9^, ^12^ These altered microbiome profiles associated with different eosinophil counts may cause distinct airway inflammation profiles in the airway which respond differently to anti‐inflammatory drugs.^13^

Here, we report a further analysis using samples from our bronchoscopy study that compared COPD eosinophil^high^ with eosinophil^low^ patients;^3^ we focus on differences in adaptive immune function that may cause altered immunity against bacteria. We investigated IgA, IgM and IgG levels using different samples and techniques, and measured B‐cell activation and bacterial opsonization.

## 2 METHODS

### 2.1 Patient recruitment and sample collection

21 eosinophil^low^ (blood count < 150/µl) and 20 eosinophil^high^ (>250/µl) COPD patients were recruited for bronchoscopy. Cut‐offs were chosen as they identified upper and lower tertiles for blood eosinophils counts in COPD patients previously studied at the Medicines Evaluation Unit, Manchester, UK. As fluctuations in blood eosinophil numbers in COPD patients are typically minor, particularly at lower blood eosinophil counts,^14^, ^15^, ^16^ the exclusion of the middle tertile helped ensure the eosinophil^high^ and eosinophil^low^ patients were distinct populations. Initial results and clinical characteristics were reported previously^3^ (Table 1). Patients with COPD >40 years old with >10 pack‐year smoking history and post‐bronchodilator forced expiratory volume in 1 second (FEV_1_)/forced vital capacity (FVC) ratio <0.7 were recruited.^2^ Patients with a previous diagnosis of childhood or adult asthma, or those with atopy demonstrated by a positive skin prick test against house dust mite extract or cat dander or grass pollen allergens (Alk‐Abello, Hørsholm Denmark) were excluded. Patients receiving oral corticosteroids or antibiotics within 6 weeks of recruitment were excluded. Bronchial biopsies, bronchoalveolar lavage and bronchial brush samples were collected; some patients were not able to tolerate collection of all samples. B cells were isolated from the blood of a healthy non‐smoking volunteer. Sample collection was approved by the local research ethics committee (NRES Committee North West – Greater Manchester South; REC Ref: 06/Q1403/156). All subjects provided written informed consent.

**TABLE 1:** Demographics and clinical characteristics of blood eosinophil^low^ and blood eosinophil^high^ patients

	Eosinophil^Low^ (*n* = 21)	Eosinophil^High^ (*n* = 20)	*P* value
Age (years)^(1)^	61.6 (5.9)	62.2 (4.4)	.70
Gender (% Male)^(3)^	57	70	.52
Current smokers (%)^(3)^	43	60	.35
Smoking pack‐years history^(1)^	41.5 (15.2)	37.7 (14.0)	.41
ICS users (*n*)^(3)^	16	11	.20
GOLD Stage 1 (*n*) ^(3)^	1	1	1.00
GOLD Stage 2 (*n*) ^(3)^	18	18	1.00
GOLD Stage 3 (*n*) ^(3)^	2	1	1.00
Post‐bronchodilator FEV_1_ (L)^(1)^	1.79 (0.43)	1.89 (0.40)	.45
Post‐bronchodilator FEV_1_ (% Predicted)^(1)^	62.4 (11.2)	65.5 (10.9)	.38
Post‐bronchodilator FVC (L)^(1)^	3.72 (1.16)	3.62 (0.87)	.77
Post‐bronchodilator FEV1/FVC ratio^(1)^	0.50 (0.1)	0.53 (0.1)	.34
Reversibility (ml)^(1)^	189.5 (183.1)	219.5 (153.8)	.57
Reversibility (%)^(1)^	13.1 (11.8)	15.3 (13.9)	.58
FeNO_50_ (ppm)^(2)^	14.8 [5.0‐30.0]	21.0 [2.2‐61.0]	.24
BMI (kg/m^2^)^(2)^	29.2 [17.9‐33.8]	25.5 [18.7‐32.2]	.11
Total SGRQ^(1)^	42.3 (15.2)	36.6 (21.3)	.37
mMRC^(2)^	1 [1‐4]	1 [0‐4]	.10
CAT^(2)^	17 [5‐35]	17 [4‐32]	.48
0 exacerbations, 12 month prior (*n*)^(3)^	12	13	.75
1 exacerbations, 12 month prior (*n*)^(3)^	4	3	1.00
≥2 exacerbations, 12 month prior (*n*)^(3)^	5	4	1.00
White blood cell count (×10^9^/L)^(1)^	7.03 (1.62)	7.57 (1.91)	.34
Blood eosinophil count (×10^9^/L)^(1)^	0.10 (0.03)	0.43 (0.15)	<.0001
Blood neutrophil count (×10^9^/L)^(1)^	4.40 (1.33)	4.25 (1.45)	.75
Blood lymphocyte count (×10^9^/L)^(1)^	1.85 (0.50)	2.14 (0.64)	.12
Blood monocyte count (×10^9^/L)^(1)^	0.61 (0.16)	0.68 (0.25)	.25
Blood basophil count (×10^9^/L)^(1)^	0.04 (0.03)	0.06 (0.02)	.03

Note

Data presented as number, %, mean (SD) or median [range]. Comparisons between Eosinophil^Low^ and Eosinophil^High^ were by: (1) *t* test; (2) Mann–Whitney or (3) Fisher's exact test.

Abbreviations: BMI, Body Mass Index; CAT, COPD Assessment Test; FEV_1_, Forced Expired Volume in first second; FVC, Forced vital capacity; GOLD, Global Initiative for Chronic Obstructive Lung Disease (FEV_1_% predicted: GOLD stage 1: ≥80%; GOLD stage 2:79%‐50%; GOLD stage 3:49%‐30%); ICS, Inhaled corticosteroid use; mMRC, modified Medical Research Council; SGRQ, St George's Respiratory Questionnaire.

### 2.2 Gene expression analysis

Bronchial epithelial brushings were collected from the lower lobes (*n* = 20 eosinophil^high^; *n* = 17 eosinophil^low^), and blood was collected into PaxGene RNA tubes (BD biosciences, Wokingham, UK). Gene expression of the immunoglobulin genes *IGHA1, IGHA2, IGHM, JCHAIN, IGHG1, IGHG2, IGHG3*, *IGHG4* and polymeric immunoglobulin receptor (*PIGR*) were assessed and genes associated with plasma cell activity, identified by literature review. These genes are listed in Table 2, including a functional description. Detailed methods are in the online data supplement.

**TABLE 2:** Bronchial epithelial gene expression of immunoglobulin regulatory mediators in eosinophil high‐ and low‐COPD patients

Gene	Function	Relative gene expression		*P*	Reference
		Eosinophil high	Eosinophil Low		
*TGFB1*	Down‐regulation of immunoglobulin	10.0 ± 1.1	9.7 ± 1.0	.38	46
*TNFSF13*	Down‐regulation of immunoglobulin	9.5 ± 0.7	9.2 ± 0.7	.16	47
*TNFSF13B*	Down‐regulation of immunoglobulin	7.1 ± 1.7	6.9 ± 1.2	.61	47
*NOS2*	Up‐regulates IgA production	9.9 ± 1.4	8.3 ± 1.6	.003	26
*LTA*	Eosinophil dependent IgA production	4.0 ± 1.1	3.0 ± 0.6	.001	48
*LTB*	Eosinophil dependent IgA production	7.0 ± 1.5	5.7 ± 1.3	.01	48
*IL1B*	Eosinophil dependent IgA production	9.5 ± 2.2	9.2 ± 1.7	.69	48
*CXCL13*	Up‐regulates immunoglobulin production	2.7 ± 2.6	3.2 ± 1.9	.49	49
*IL5*	Up‐regulates IgA production	1.8 ± 1.4	2.0 ± 0.8	.51	50

### 2.3 BAL Immunoglobulin assessment

BAL was successfully collected from *n* = 15 eosinophil^high^ and *n* = 15 eosinophil^low^. Levels of IgG1 and IgG2 in BAL fluid were determined by ELISA (ThermoFisher). Secretory IgA levels were assessed by ELISA (Demeditec). IgA and IgM levels have been previously assessed in the same BAL samples by multiplex assay (Myriad RBM).^3^ Levels of immunoglobulins were normalized to patient‐specific BAL urea concentrations,^17^ measured by colorimetric assay (Biovision Inc). The use of urea for normalization is not universally accepted,^18^ so for completeness, we also present non‐normalized results in the online supplement.

### 2.4 Bronchial biopsy Immunohistochemistry

Formalin‐fixed, paraffin‐embedded bronchial biopsies were assessed for levels of B cells and plasma cells, along with expression of IgA2, IgG1, IgG2 and IgM by immunohistochemistry. Detailed methods are in the online data supplement.

### 2.5 In vitro B‐cell treatment

B cells from the blood of a healthy non‐smoking volunteer were exposed to BAL fluid from eosinophil^high^ and eosinophil^low^ patients. Healthy donor B cells were preferred over COPD B cells, which may be dysfunctional giving data that is difficult to interpret. The effect of BAL on X‐box‐binding protein 1 (*XBP1*) gene expression was assessed by quantitative PCR. Detailed methods are in the online data supplement.

### 2.6 NTHi‐specific immunoglobulin binding assay

Methodology for immunoglobulin‐specific opsonization of non‐typeable haemophilus influenza (NTHi) was adapted from Staples et al^19^ Detailed methods are in the online data supplement.

### 2.7 Statistical analysis

All results were assessed using Graphpad Prism version 7.04. Comparisons between eosinophil^high^ and eosinophil^low^ were by *t* test or Mann‐Whitney test, with distribution of data assessed by D'Agostino & Pearson normality test.

## 3 RESULTS

### 3.1 Subjects

Clinical characteristics for the 21 eosinophil^low^ and 20 eosinophil^high^ COPD patients are summarized in Table 1. Lung function, proportion of patients using ICS, symptoms and exacerbation rates were similar between the groups, with the majority of patients being GOLD stage 2. No history of immunodeficiency was reported for any patient, and blood immunoglobulin gene expression levels were similar in eosinophil^high^ and eosinophil^low^ patients, as were serum IgA and IgM levels (Table S1). Blood basophil numbers were higher in eosinophil^high^ compared with eosinophil^low^ patients.

### 3.2 IgA and IgM BAL protein and epithelial gene expression

**FIGURE 1:**
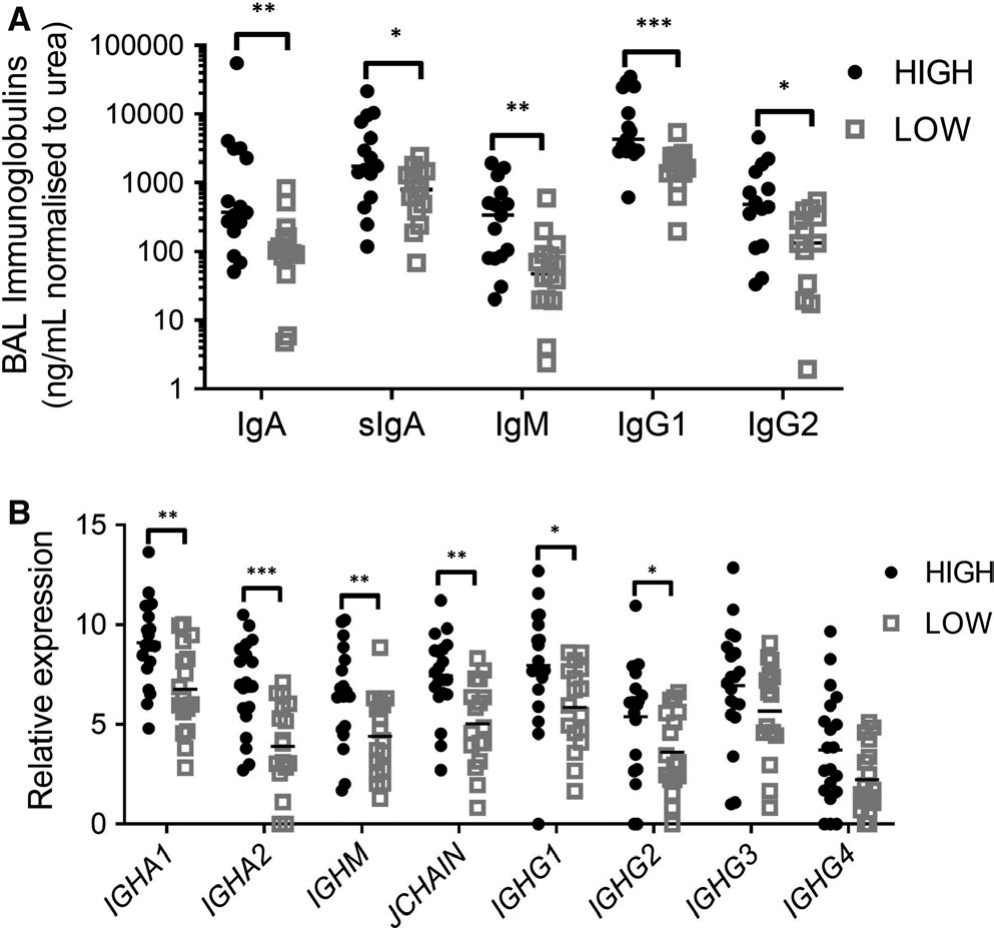
Immunoglobulin levels in BAL (A) and gene expression in bronchial epithelial cells (B) from eosinophil^high^ and eosinophil^low^ COPD patients. A, IgA and IgM levels in BAL were measured by Myriad RBM assay, while secretory IgA, IgG1 and IgG2 were assessed by ELISA. Immunoglobulin concentrations have been normalized to BAL urea levels. B, Epithelial gene expressions of immunoglobulins were assessed by RNA‐Seq. Comparisons between eosinophil^high^ eosinophil^low^ patients were by Mann‐Whitney for BAL and *t* test for epithelial gene expression: *P < .05; **P < .01; ***P < .001. Bars illustrated median (A) or mean (B) values

Previously, we briefly reported that COPD eosinophil^high^ patients had higher absolute BAL IgA and IgM protein levels, compared with eosinophil^low^ patients (Figure S1A,B).^3^ BAL recovery rates vary between patients, causing variable dilution effects. We now present BAL immunoglobulin results normalized to patient‐specific BAL urea levels (Figure 1A); BAL IgA and IgM protein levels remained higher in eosinophil^high^ compared to eosinophil^low^ patients. Immunoglobulin gene expression in epithelial brushings from the same patients (Figure 1B) demonstrated higher expression of genes for the IgA and IgM heavy chains, and the J chain that enables IgA and IgM polymerization, in the eosinophil^high^ COPD patients. Secretory IgA levels in BAL from eosinophil^high^ patients were higher compared with eosinophil^low^ patients (Figure 1A; Figure S1C).

### 3.3 IgG BAL protein and epithelial gene expression

Bronchial epithelial gene expression levels of *IGHG1* and *IGHG2* were greater in COPD eosinophil^high^ patients (Figure 1B). The mean levels of *IGHG3* and *IGHG4* gene expression were also higher in the eosinophil^high^ group, but did not reach statistical significance (Figure 1B). We then measured IgG1 and IgG2 levels in BAL; the levels of these immunoglobulins were higher in BAL from eosinophil^high^ compared to eosinophil^low^ patients (median IgG1: 4340 vs 1772 ng/ml respectively, *P* = .0003; median IgG2: 488 vs 133 ng/ml respectively, *P* = .0186; Figure 1A with urea normalized results and Figure S1D,E with non‐normalized results).

### 3.4 Immunoglobulin immunohistochemistry

**FIGURE 2:**
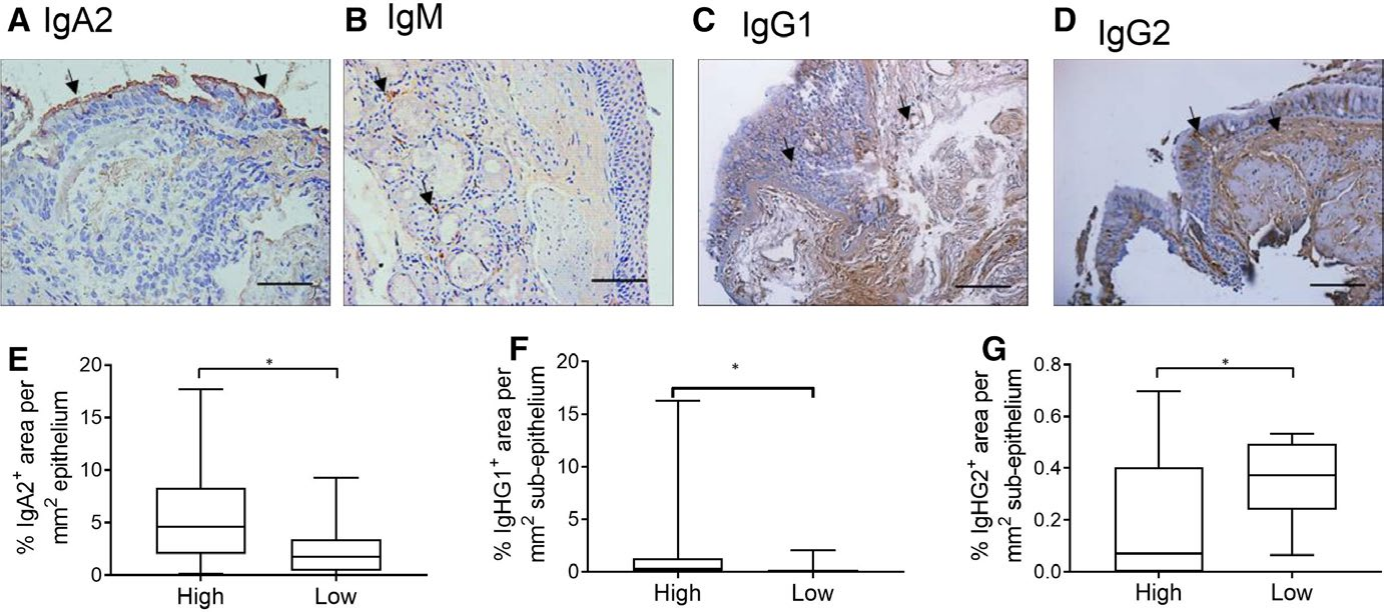
Immunohistochemical analysis of immunoglobulin expression in bronchial biopsies from eosinophil^high^ and eosinophil^low^ patients. Bronchial biopsies were formalin‐fixed, paraffin‐embedded. Tissue sections were stained for IgA2 (A), IgM (B), IgG1 (C) and IgG2 (D). Images represent typical staining for each immunoglobulin, with arrows identifying examples of positive areas; size bars illustrate 100μm. Comparisons of epithelial IgA2 (E), and subepithelial IgG1 (F) and IgG2 (G) between eosinophil^high^ and eosinophil^low^ were by Mann‐Whitney test: *P < .05. Box and whisker plots represent median, interquartile range and range

IgA was predominantly found on the apical surface of the bronchial epithelium (Figure 2A). The percentage of the epithelium immunoreactive for IgA was significantly higher in the eosinophil^high^ patients compared with the eosinophil^low^ patients (Figure 2E).

Remodelled airways in COPD patients have limited apical IgA expression.^20^ This has been associated with a localized reduction in polymeric immunoglobulin receptor (pIgR) protein and gene expression, resulting in flawed IgA epithelial translocation. *PIGR* expression in epithelial brush samples was similar between the groups (eosinophil^high^: 15.78 ± 0.55; eosinophil^low^: 15.50 ± 0.57; *P* = .129), suggesting that the observed differences in apical IgA staining are unlikely due to differences in translocation.

The majority of bronchial biopsies were negative for epithelial IgM expression. Expression was observed in the submucosal bronchial glands (Figure 2B). Approximately 40% of biopsies contained submucosal glands making comparisons between groups difficult with small sample sizes.

IgG1 and IgG2 expression was identified in both the epithelium and subepithelium (Figure 2C,D). IgG1 subepithelium expression was significantly higher in eosinophil^high^ patients (Figure 2F), while subepithelial IgG2 expression was significantly higher in the eosinophil^low^ patients (Figure 2G); epithelial expression of IgG1 and IgG2 showed similar trends (Figure S2; *P* = .08 and *P* = .05, respectively).

### 3.5 B‐cell numbers and activity

**FIGURE 3:**
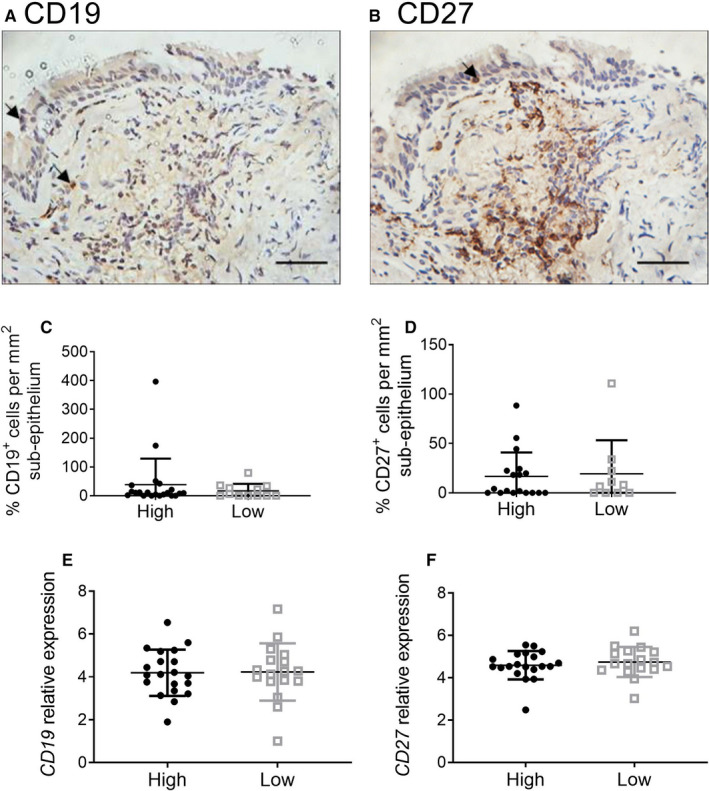
Quantification of CD19^+^ B cells and CD27^+^ plasma cells in bronchial tissue from eosinophil^high^ and eosinophil^low^ patients. Bronchial biopsies were formalin‐fixed, paraffin‐embedded. Tissue sections were stained for CD19 (A &C) and CD27 (B & D) with results presented as the number of positive cells per mm^2^ of subepithelium. Images A and B represent typical staining; size bars illustrate 100 μm. RNA was extracted and sequenced from bronchial brush samples. Data are presented as relative gene expression for CD19 (E) and CD27 (F). Comparisons between eosinophil^high^ and eosinophil^low^ were by Mann‐Whitney test: all non‐significant. Bars represent median values with interquartile range

Differences in immunoglobulin levels may be due to different B cells or plasma cell numbers. We assessed levels of CD19^+^ B cells and CD27^+^ plasma cells in bronchial biopsies by immunohistochemistry (Figure 3A,B). B cells and plasma cell numbers were similar between the eosinophil^high^ and eosinophil^low^ groups (Figure 3C,D). Similar gene expression of *CD19* and *CD27* in bronchial epithelial brush samples supported these findings (Figure 3E,F).

**FIGURE 4:**
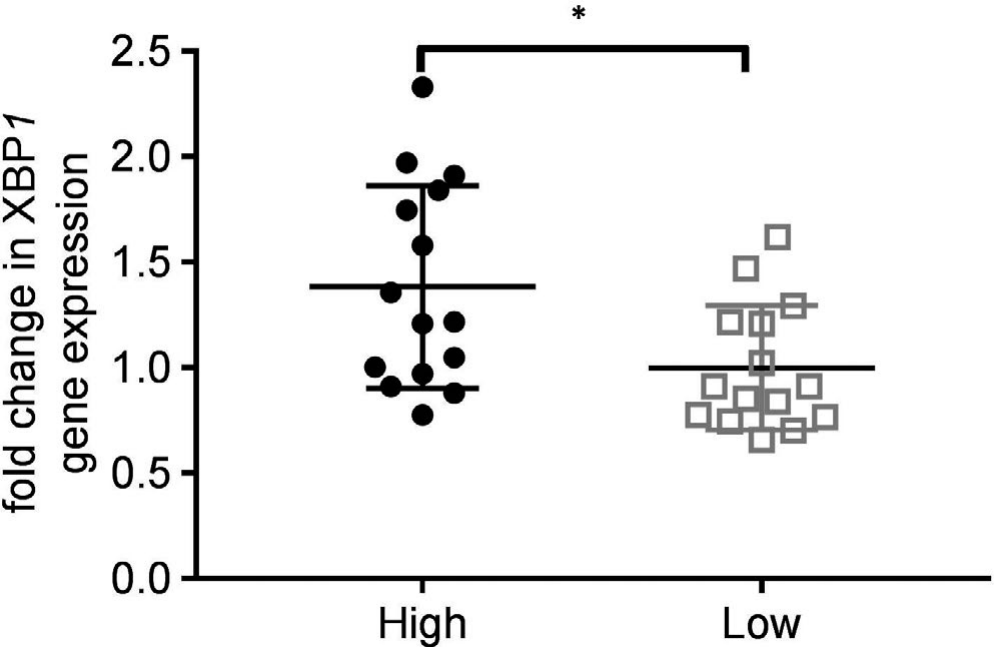
Activation of B‐cell *XBP1* gene expression following treatment with BAL from eosinophil^high^ and eosinophil^low^ patients. B cells were isolated from the blood of a healthy subject, and then treated with BAL fluid for 72 h. Gene expression of *XBP1* was assessed by quantitative PCR, with data presented as fold change compared to untreated B cells. Comparison between eosinophil^high^ and eosinophil^low^ was by *t* test: *P < .05. Bars represent mean values ± standard deviation

We treated healthy B cells with BAL fluid from eosinophil^high^ and eosinophil^low^ patients to assess B‐cell activation. Cells treated with eosinophil^high^ BAL expressed higher levels of *XBP1*, a key regulator of plasma cell immunoglobulin production (Figure 4). We compared bronchial epithelial gene expression of mediators that influence plasma cell activity (Table 2). Levels of *NOS2* and the lymphotoxins *LTA* and *LTB* were significantly lower in eosinophil^low^ patients.

### 3.6 NTHi opsonization

**FIGURE 5:**
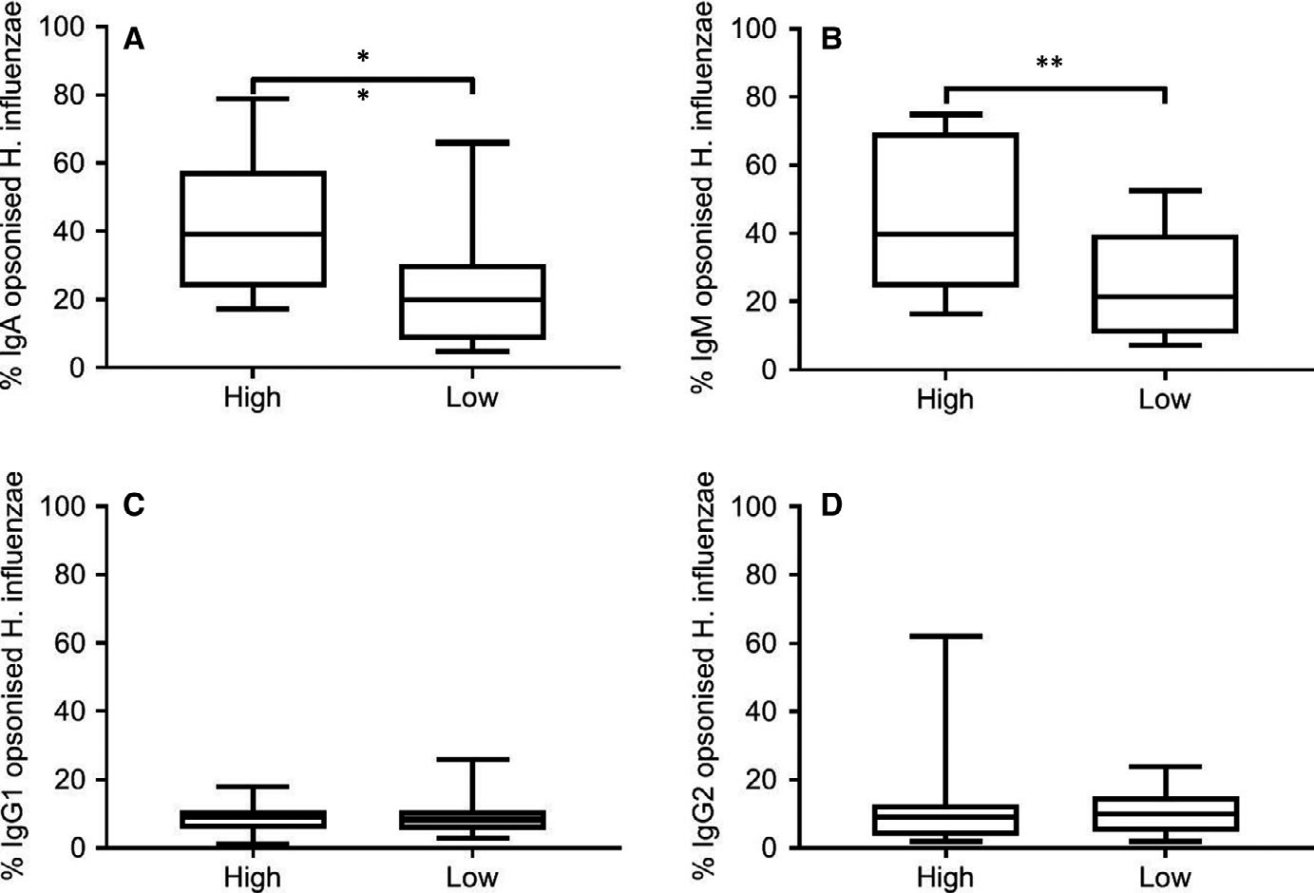
Immunoglobulin‐specific opsonization of NTHi with BAL from eosinophil^high^ and eosinophil^low^ patients. NTHi bacteria was treated with BAL for 30 min and then labelled with fluorescent antibodies against IgA, IgM, IgG1 and IgG2 to allow levels of opsonization to be assessed by flow cytometry. Data are presented as the percentage of bacterial cells showing positive opsonization. Comparisons between eosinophil^high^ and eosinophil^low^ were by Mann‐Whitney test: **P < .01. Bars represent median values with interquartile range

Reduced immunoglobulin levels in eosinophil^low^ COPD patients may confer reduced anti‐bacterial activity. Immunoglobulin‐specific NTHi opsonization experiments were carried out using BAL fluid. The results showed that approximately twice as many bacteria were opsonized with IgA and IgM using BAL fluid from eosinophil^high^ compared to eosinophil^low^ patients (Figure 5), indicating lower levels of NTHi‐specific IgA and IgM antibodies in eosinophil^low^ patients. Levels of NTHi‐specific IgG1 and IgG2 opsonization were similar between groups.

## 4 DISCUSSION

We have demonstrated higher IgA, secretory IgA, IgM and IgG1 levels in COPD eosinophil^high^ compared with eosinophil^low^ patients. These results were consistent when immunoglobulins were measured by 3 different methods; BAL protein measurement, bronchial epithelial gene expression and bronchial mucosal immunohistochemistry. Furthermore, BAL supernatants from COPD eosinophil^high^ patients caused greater NTHi opsonization and B‐cell activation. Overall, these findings indicate reduced anti‐proteobacterial immunity in COPD eosinophil^low^ patients.

Pneumonia risk is greater in COPD patients with lower blood eosinophil counts.^10^, ^21^ Lower airway eosinophil numbers are associated with increased presence of colonizing bacteria in stable COPD patients, and ICS use was associated with an increase in bacterial load only in patients with lower eosinophils.^6^, ^7^, ^8^, ^9^ Overall, these previous findings suggest that COPD patients with lower eosinophil counts have a different susceptibility to bacterial infection.^22^ Our data showing lower immunoglobulin levels and activity in COPD eosinophil^low^ patients provide a potential mechanistic explanation for these observations. The risk of pneumonia in COPD patients is associated with certain clinical characteristics, such as previous pneumonia events, older age and lower body mass index.^2^ The use of ICS in such individuals may increase pneumonia risk further.^2^ Overall, there appear to be complex interactions between clinical characteristics, blood eosinophil counts and ICS use that influence pneumonia risk in COPD patients.^22^

COPD and asthma patients with lower airway eosinophils have greater levels of proteobacteria, including Haemophilus.^9^, ^11^, ^12^ NTHi is found in the airways of COPD patients, both at stable state and during exacerbation.^12^, ^13^, ^23^ NTHi strains isolated from COPD patients have enriched virulence factors, including genes associated with iron uptake, immune evasion, adherence and oxidative stress counter‐measures.^24^, ^25^ In addition to these mechanisms that can potentiate or prolong NTHi activity, we now show lower antibody‐mediated immunity against NTHi in COPD eosinophil^low^ patients.

IgA is the predominant immunoglobulin found in the human lung, with 80% of mucosal plasma cells being IgA^+^.^26^ BAL IgA levels from mild/moderate COPD patients are higher compared with controls.^19^ However, Du et al reported lower BAL IgA levels in severe/very severe patients compared with controls.^27^ We show lower BAL IgA levels in COPD patients with lower eosinophil levels. IgA is produced by subepithelial plasma cells before being transported through the epithelial layer in a complex with polymeric immunoglobulin receptor (pIgR).^28^ Released secretory IgA (sIgA) coats the mucosal surface, providing protection against microbes. The remodelled epithelium in COPD patients has reduced pIgR expression, causing decreased mucosal IgA.^29^, ^30^ Areas of decreased IgA show localized increases in inflammatory cells and submucosal invasion by both bacteria and viruses.^20^, ^29^, ^30^ We observed that eosinophil^low^ COPD patients had lower mucosal IgA expression, which was not due to altered epithelial IgA translocation, as *PIGR* expression was similar between groups. Our findings may be due to reduced B‐cell activity in the eosinophil^low^ patients. Lower BAL IgA levels in COPD have previously been associated with increased NTHi colonization,^19^ supporting a link between eosinophils, immunoglobulins and microbiome.

Eosinophil^low^ patients also had lower levels of NTHi‐specific IgA, which induced lower levels of bacterial opsonization. Staples et al showed reduced bacterial opsonization, following BAL supernatant exposure, results in lower levels of NTHi phagocytosis ^19^; this mechanism may contribute to the association between a low airway eosinophils phenotype and increased bacterial colonization.^6^, ^7^, ^8^, ^9^

IgG1, IgG2 and IgG3 levels are elevated in BAL from COPD patients compared with controls, but IgG1 and NTHi‐specific IgG1 levels are lower in patients colonized by NTHi.^19^ IgG1 is the most abundant form of IgG. There were lower BAL levels of total IgG1 in COPD eosinophil^low^ patients compared with eosinophil^high^ patients, but we did not show a difference between groups for NTHi‐specific IgG1 antibodies. Further work is required to see if eosinophil^low^ patients have impaired IgG responses against other bacterial pathogens, such as Moraxella catarrhalis and Streptococcus pneumonia.^6^ Although *IGHG2* expression was lower in eosinophil^low^ patients, higher protein levels were retained in the subepithelium. This may be due to IgG2 being actively retained within the tissue to combat subepithelial bacterial colonization associated with lower apical IgA expression.

Lymphoid follicles act as focal centres for antigen presentation and adaptive immune responses; increased numbers of B‐cell rich lymphoid follicles are associated with COPD severity.^31^ IgM^+^ plasma cells are the predominant B‐cell found in airway lymphoid follicles. BAL IgM levels were reported to be higher in COPD patients compared to controls.^19^, ^32^ We have shown lower levels of IgM BAL protein and epithelial gene expression in eosinophil^low^ compared with eosinophil^high^ COPD patients. It is probable that these epithelial gene expression results are due to bronchial brush samples contain a mixture of epithelial and other cell types including B cells.

We were unable to determine if the distribution of tissue IgM differed between the eosinophil^high^ and eosinophil^low^ groups. Immunohistochemical analysis showed that IgM was predominantly found in mucosal glands. Previous work has shown that immunoglobulins produced in glands do not distribute across the airway mucosal surface, but are localized to mucous plugs.^27^ Like IgA, we showed that eosinophil^low^ COPD patients had reduced levels of NTHi‐specific IgM in BAL compared with eosinophil^high^ patients.

B‐cell and plasma cell numbers are increased in both the large and small airways of COPD patients compared to controls.^33^, ^34^ A recent study reported that lymphoid follicles in severe COPD patients have increased IgA^+^ plasma cells.^32^ We found no differences in B‐cell or plasma cell numbers in biopsies from eosinophil^high^ and eosinophil^low^ patients. However, BAL fluid from eosinophil^low^ patients induced lower levels of *XBP1* expression than BAL from eosinophil^high^ patients. The transcription factor Xbp1 is required for plasma cell differentiation and is essential for high levels of antibody secretion,^35^ and our findings suggest that the airways of eosinophil^low^ patients may be less primed to stimulate immunoglobulin production. In smoke‐exposed mice, efficient clearance of NTHi has been shown to be B‐cell dependent and relates to levels of NTHi‐specific IgA.^36^

The epithelial gene expression of *NOS2* and lymphotoxins *LTA* and *LTB* was lower in the eosinophil^low^ group; these findings are discussed further in the online supplement.

We previously reported higher BAL IL‐5 levels in eosinophil^high^ patients.^3^ This cytokine is important for eosinophil maturation, activation and survival, and stimulates IgA production by plasma cells in mice,^37^ although this has not been shown in humans. A Phase 2a placebo‐controlled, randomized clinical trial in COPD patients with sputum eosinophilia showed that the anti‐IL‐5 receptor monoclonal antibody, benralizumab, caused near‐complete depletion of blood and sputum eosinophils.^38^ Phase 3 clinical trials with benralizumab did not show efficacy in the primary analysis of exacerbation rate reduction,^39^ but pre‐specified subgroup analysis demonstrated that benralizumab reduced exacerbation rates in patients with blood eosinophil levels ≥220 cells/µl, ≥3 exacerbations in the prior year and using inhaled triple therapy.^39^ This suggests that increased eosinophil activity has a mechanistic involvement in disease pathophysiology in a subgroup of COPD patients with certain clinical characteristics plus higher blood eosinophil counts.

The Phase III COPD clinical programs conducted with the anti‐eosinophil therapies benralizumab and mepolizumab showed no evidence of increased exacerbations or bacterial infections compared to placebo,^40^, ^41^ supporting that IL‐5 and/or eosinophils are not directly responsible for the higher immunoglobulin levels seen in our eosinophil^high^ COPD patients. Furthermore, in the Phase II COPD trial, sputum microbiome analysis demonstrated no increase in airway bacterial load with benralizumab treatment, but rather a reduction in Streptococcus pneumonia levels, with no effect on Haemophilus influenzae.^38^ The authors proposed that Streptococcus pneumonia reduction may be due to macrophages being redirected from eosinophil efferocytosis towards bacterial phagocytosis after eosinophil depletion. Additionally, eosinophils have no direct anti‐bacterial activity against Streptococcus pneumonia and Haemophilus influenza.^38^ Overall, the eosinophil itself appears to have little direct anti‐bacterial activity, but here we show that eosinophil^low^ COPD patients display differences in B‐cell activity and immunoglobulin levels that may cause differences in microbiome.

We have recently shown higher airway IL‐13 levels in the same group of eosinophil^high^ (versus eosinophil^low^) patients in this report.^4^ IL‐13 promotes humoral responses.^42^ It is possible that the T2 environment present in the airways of eosinophil^high^ COPD patients facilitates better anti‐bacterial defence through mechanisms such as IL‐13‐stimulated B‐cell activation.

Hypereosinophilic syndrome commonly involves the lungs (63% of patients).^43^ The airways from chronic eosinophilic pneumonia (CEP) patients contain many more eosinophils compared to eosinophil^high^ COPD patients; for example, median BAL eosinophil percentage in CEP is 52% and 0.75% in eosinophil^high^ COPD.^3^, ^44^ However, like eosinophil^high^ COPD, CEP patients also have elevated airway type 2 inflammation ^44^ and increased BAL levels of IgA, IgM and IgG.^45^

We did not include healthy controls, as it has already been reported that immunoglobulin expression levels are higher in mild COPD patients compared with controls,^19^ but more severe patients have lower secretory IgA levels.^27^ We cannot state if the immunoglobulin levels observed here are elevated or decreased compared to normal levels, but can state that immunoglobulin levels are higher in eosinophil^high^ relative to the eosinophil^low^ patients. There is growing evidence that low eosinophil numbers are associated with bacterial colonization.^6^, ^7^, ^8^, ^10^ A limitation is that we did not assess bacteriology in the current study, preventing comparisons between immunoglobulin levels and bacterial load. We had few severe COPD patients, as these individuals are practically more difficult to bronchoscope for research. The sample size of 41 subjects was modest, but sufficient to observe consistent biological differences between groups.

In conclusion, we have shown differences in adaptive immunity in COPD eosinophil^low^ patients compared with COPD eosinophil^high^ patients, associated with altered anti‐proteobacterial immunity. These results can explain differences in the airway microbiome associated with eosinophil counts that have been reported.^6^, ^7^, ^8^, ^9^, ^10^

## CONFLICT OF INTEREST

DS reports personal fees from AstraZeneca, personal fees from Boehringer Ingelheim, personal fees from Chiesi, personal fees from Cipla, personal fees from Genentech, personal fees from GlaxoSmithKline, personal fees from Glenmark, personal fees from Gossamerbio, personal fees from Menarini, personal fees from Mundipharma, personal fees from Novartis, personal fees from Peptinnovate, personal fees from Pfizer, personal fees from Pulmatrix, personal fees from Theravance, personal fees from Verona. SS, T‐HP and PN are employees of AstraZeneca and also hold stock in AstraZeneca. T. Southworth, AH, UK, JL, JD and T. Scott have no conflicts to report.

## AUTHOR CONTRIBUTION

**Thomas Southworth:** Conceptualization (lead); Formal analysis (lead); Funding acquisition (supporting); Investigation (equal); Methodology (equal); Project administration (lead); Resources (lead); Supervision (lead); Visualization (lead); Writing‐original draft (lead); Writing‐review & editing (equal). **Andrew Higham:** Conceptualization (supporting); Formal analysis (supporting); Investigation (equal); Methodology (equal); Project administration (supporting); Supervision (supporting); Visualization (supporting); Writing‐original draft (supporting); Writing‐review & editing (equal). **Umme Kolsum:** Conceptualization (supporting); Formal analysis (supporting); Funding acquisition (supporting); Investigation (equal); Methodology (supporting); Project administration (supporting); Resources (lead); Supervision (supporting); Visualization (supporting); Writing‐review & editing (equal). **Jian Li:** Formal analysis (supporting); Investigation (equal); Methodology (equal); Visualization (supporting); Writing‐original draft (supporting); Writing‐review & editing (equal). **Thomas Scott:** Formal analysis (supporting); Investigation (equal); Methodology (equal); Visualization (supporting); Writing‐original draft (supporting); Writing‐review & editing (equal). **Josiah Dungwa:** Formal analysis (supporting); Investigation (equal); Methodology (supporting); Visualization (supporting); Writing‐review & editing (equal). **Sriram Sridhar:** Formal analysis (supporting); Investigation (equal); Methodology (equal); Writing‐original draft (supporting); Writing‐review & editing (equal). **Tuyet‐Hang Pham:** Formal analysis (supporting); Investigation (supporting); Methodology (equal); Writing‐original draft (supporting); Writing‐review & editing (equal). **Paul Newbold:** Conceptualization (lead); Funding acquisition (lead); Investigation (equal); Project administration (supporting); Supervision (supporting); Writing‐review & editing (equal). **Dave Singh:** Conceptualization (lead); Funding acquisition (lead); Project administration (supporting); Resources (lead); Supervision (lead); Writing‐original draft (lead); Writing‐review & editing (equal).

## Supporting information

Supplementary Material

## Data Availability

The data that support the findings of this study are available from the corresponding author upon reasonable request.

## References

[1] Siddiqui SH , Guasconi A , Vestbo J , et al. Blood eosinophils: a biomarker of response to extrafine beclomethasone/formoterol in chronic obstructive pulmonary disease. Am J Respir Crit Care Med. 2015;192:523‐525. 26051430 10.1164/rccm.201502-0235LE PMC4595668

[2] Singh D , Agusti A , Anzueto A , et al. Global strategy for the diagnosis, management, and prevention of chronic obstructive lung disease: the GOLD Science Committee Report 2019. Eur Respir J. 2019;53:1900164. 30846476 10.1183/13993003.00164-2019

[3] Kolsum U , Damera G , Pham TH , et al. Pulmonary inflammation in patients with chronic obstructive pulmonary disease with higher blood eosinophil counts. J Allergy Clin Immunol. 2017;140:1181‐1184.e7. 28506852 10.1016/j.jaci.2017.04.027

[4] Higham A , Beech A , Wolosianka S , et al. Type 2 inflammation in eosinophilic chronic obstructive pulmonary disease. Allergy. 2020 https://onlinelibrary.wiley.com/doi/10.1111/all.14661 10.1111/all.14661 PMC8247000 33206402

[5] Flood‐Page P , Menzies‐Gow A , Phipps S , et al. Anti‐IL‐5 treatment reduces deposition of ECM proteins in the bronchial subepithelial basement membrane of mild atopic asthmatics. J Clin Invest. 2003;112:1029‐1036. 14523040 10.1172/JCI17974 PMC198522

[6] Kolsum U , Donaldson GC , Singh R , et al. Blood and sputum eosinophils in COPD; relationship with bacterial load. Respir Res. 2017;18:88. 28482840 10.1186/s12931-017-0570-5 PMC5422866

[7] Contoli M , Pauletti A , Rossi MR , et al. Long‐term effects of inhaled corticosteroids on sputum bacterial and viral loads in COPD. Eur Respir J. 2017;50:1700451. 28982774 10.1183/13993003.00451-2017

[8] Kim VL , Coombs NA , Staples KJ , et al. Impact and associations of eosinophilic inflammation in COPD: analysis of the AERIS cohort. Eur Respir J. 2017;50:1700853. 29025891 10.1183/13993003.00853-2017

[9] Beech AS , Lea S , Kolsum U , et al. Bacteria and sputum inflammatory cell counts; a COPD cohort analysis. Respir Res. 2020;21:289. 33131502 10.1186/s12931-020-01552-4 PMC7603729

[10] Martinez‐Garcia MA , Faner R , Oscullo G , et al. Inhaled steroids, circulating eosinophils, chronic airway infection and pneumonia risk in chronic obstructive pulmonary disease: a network analysis. Am J Respir Crit Care Med. 2020;201:1078‐1085. 31922913 10.1164/rccm.201908-1550OC

[11] Ghebre MA , Pang PH , Diver S , et al. Biological exacerbation clusters demonstrate asthma and chronic obstructive pulmonary disease overlap with distinct mediator and microbiome profiles. J Allergy Clin Immunol. 2018;141:2027‐2036.e12. 29709671 10.1016/j.jaci.2018.04.013 PMC5986707

[12] Wang Z , Bafadhel M , Haldar K , et al. Lung microbiome dynamics in COPD exacerbations. Eur Respir J. 2016;47:1082‐1092. 26917613 10.1183/13993003.01406-2015

[13] Wang Z , Maschera B , Lea S , et al. Airway host‐microbiome interactions in chronic obstructive pulmonary disease. Respir Res. 2019;20:113. 31170986 10.1186/s12931-019-1085-z PMC6555748

[14] Long GH , Southworth T , Kolsum U , et al. The stability of blood Eosinophils in chronic obstructive pulmonary disease. Respir Res. 2020;21:15. 31924207 10.1186/s12931-020-1279-4 PMC6954589

[15] Southworth T , Beech G , Foden P , Kolsum U , Singh D . The reproducibility of COPD blood eosinophil counts. Eur Respir J. 2018;52:1800427 10.1183/13993003.00427-2018 29724922

[16] Singh D , Bafadhel M , Brightling CE , et al. Blood eosinophil counts in clinical trials for chronic obstructive pulmonary disease. Am J Respir Crit Care Med. 2020;202:660‐671. 32186896 10.1164/rccm.201912-2384PP PMC7462391

[17] Rennard SI , Basset G , Lecossier D , et al. Estimation of volume of epithelial lining fluid recovered by lavage using urea as marker of dilution. J Appl Physiol. 1985;1986(60):532‐538. 10.1152/jappl.1986.60.2.532 3512509

[18] Ward C , Duddridge M , Fenwick J , et al. Evaluation of albumin as a reference marker of dilution in bronchoalveolar lavage fluid from asthmatic and control subjects. Thorax. 1993;48:518‐522. 8322239 10.1136/thx.48.5.518 PMC464506

[19] Staples KJ , Taylor S , Thomas S , et al. Relationships between Mucosal Antibodies, Non‐Typeable Haemophilus influenzae (NTHi) Infection and Airway Inflammation in COPD. PLoS One. 2016;11:e0167250. 27898728 10.1371/journal.pone.0167250 PMC5127575

[20] Polosukhin VV , Cates JM , Lawson WE , et al. Bronchial secretory immunoglobulin a deficiency correlates with airway inflammation and progression of chronic obstructive pulmonary disease. Am J Respir Crit Care Med. 2011;184:317‐327. 21512171 10.1164/rccm.201010-1629OC PMC3265275

[21] Pavord ID , Lettis S , Anzueto A , Barnes N . Blood eosinophil count and pneumonia risk in patients with chronic obstructive pulmonary disease: a patient‐level meta‐analysis. Lancet Respir Med. 2016;4:731‐741. 27460163 10.1016/S2213-2600(16)30148-5

[22] Dransfield MT , Singh D . Predicting pneumonia in chronic obstructive pulmonary disease. Have we unraveled the network of risks? Am J Respir Crit Care Med. 2020;201:1026‐1027. 32011902 10.1164/rccm.202001-0132ED PMC7193853

[23] Mayhew D , Devos N , Lambert C , et al. Longitudinal profiling of the lung microbiome in the AERIS study demonstrates repeatability of bacterial and eosinophilic COPD exacerbations. Thorax. 2018;73:422‐430. 29386298 10.1136/thoraxjnl-2017-210408 PMC5909767

[24] Zhang L , Xie J , Patel M , et al. Nontypeable Haemophilus influenzae genetic islands associated with chronic pulmonary infection. PLoS One. 2012;7:e44730. 22970300 10.1371/journal.pone.0044730 PMC3435294

[25] Qu J , Lesse AJ , Brauer AL , Cao J , Gill SR , Murphy TF . Proteomic expression profiling of Haemophilus influenzae grown in pooled human sputum from adults with chronic obstructive pulmonary disease reveal antioxidant and stress responses. BMC Microbiol. 2010;10:162. 20515494 10.1186/1471-2180-10-162 PMC2887450

[26] Tezuka H , Abe Y , Iwata M , et al. Regulation of IgA production by naturally occurring TNF/iNOS‐producing dendritic cells. Nature. 2007;448:929‐933. 17713535 10.1038/nature06033

[27] Du RH , Richmond BW , Blackwell TS Jr , et al. Secretory IgA from submucosal glands does not compensate for its airway surface deficiency in chronic obstructive pulmonary disease. Virchows Arch. 2015;467:657‐665. 26432569 10.1007/s00428-015-1854-0 PMC5081073

[28] Brandtzaeg P . Mucosal immunity: induction, dissemination, and effector functions. Scand J Immunol. 2009;70:505‐515. 19906191 10.1111/j.1365-3083.2009.02319.x

[29] Pilette C , Godding V , Kiss R , et al. Reduced epithelial expression of secretory component in small airways correlates with airflow obstruction in chronic obstructive pulmonary disease. Am J Respir Crit Care Med. 2001;163:185‐194. 11208645 10.1164/ajrccm.163.1.9912137

[30] Polosukhin VV , Richmond BW , Du RH , et al. Secretory IgA deficiency in individual small airways is associated with persistent inflammation and remodeling. Am J Respir Crit Care Med. 2017;195:1010‐1021. 27911098 10.1164/rccm.201604-0759OC PMC5422646

[31] Hogg JC , Chu F , Utokaparch S , et al. The nature of small‐airway obstruction in chronic obstructive pulmonary disease. The New England journal of medicine. 2004;350:2645‐2653. 15215480 10.1056/NEJMoa032158

[32] Ladjemi MZ , Martin C , Lecocq M , et al. Increased IgA expression in lung lymphoid follicles in severe chronic obstructive pulmonary disease. Am J Respir Crit Care Med. 2019;199:592‐602. 30339768 10.1164/rccm.201802-0352OC

[33] Gosman MM , Willemse BW , Jansen DF , et al. Increased number of B‐cells in bronchial biopsies in COPD. Eur Respir J. 2006;27:60‐64. 16387936 10.1183/09031936.06.00007005

[34] Zhu J , Qiu Y , Valobra M , et al. Plasma cells and IL‐4 in chronic bronchitis and chronic obstructive pulmonary disease. Am J Respir Crit Care Med. 2007;175:1125‐1133. 17322111 10.1164/rccm.200602-161OC

[35] Gass JN , Gunn KE , Sriburi R , Brewer JW . Stressed‐out B cells? Plasma‐cell differentiation and the unfolded protein response. Trends Immunol. 2004;25:17‐24. 14698280 10.1016/j.it.2003.11.004

[36] Gaschler GJ , Zavitz CC , Bauer CM , Stampfli MR . Mechanisms of clearance of nontypeable Haemophilus influenzae from cigarette smoke‐exposed mouse lungs. Eur Respir J. 2010;36:1131‐1142. 20413532 10.1183/09031936.00113909

[37] Schoenbeck S , McKenzie DT , Kagnoff MF . Interleukin 5 is a differentiation factor for IgA B cells. Eur J Immunol. 1989;19:965‐969. 2787753 10.1002/eji.1830190602

[38] George L , Wright A , Mistry V , et al. Sputum *Streptococcus pneumoniae* is reduced in COPD following treatment with benralizumab. Int J Chron Obstruct Pulmon Dis. 2019;14:1177‐1185. 31239655 10.2147/COPD.S198302 PMC6559763

[39] Criner GJ , Celli BR , Singh D , et al. Predicting response to benralizumab in chronic obstructive pulmonary disease: analyses of GALATHEA and TERRANOVA studies. *Lancet* . Respir Med. 2020;8:158‐170. 10.1016/S2213-2600(19)30338-8 31575508

[40] Criner GJ , Celli BR , Brightling CE , et al. Benralizumab for the prevention of COPD exacerbations. N Engl J Med. 2019;381:1023‐1034. 31112385 10.1056/NEJMoa1905248

[41] Pavord ID , Chanez P , Criner GJ , et al. Mepolizumab for eosinophilic chronic obstructive pulmonary disease. N Engl J Med. 2017;377:1613‐1629. 28893134 10.1056/NEJMoa1708208

[42] Bost KL , Holton RH , Cain TK , Clements JD . In vivo treatment with anti‐interleukin‐13 antibodies significantly reduces the humoral immune response against an oral immunogen in mice. Immunology. 1996;87:633‐641. 8675220 10.1046/j.1365-2567.1996.502574.x PMC1384144

[43] Dulohery MM , Patel RR , Schneider F , Ryu JH . Lung involvement in hypereosinophilic syndromes. Respir Med. 2011;105:114‐121. 21036585 10.1016/j.rmed.2010.09.011

[44] Miyazaki E , Nureki S , Fukami T , et al. Elevated levels of thymus‐ and activation‐regulated chemokine in bronchoalveolar lavage fluid from patients with eosinophilic pneumonia. Am J Respir Crit Care Med. 2002;165:1125‐1131. 11956056 10.1164/ajrccm.165.8.2106110

[45] Boomars KA , van Velzen‐Blad H , Mulder PG , Koenderman L , Lammers JW , van den Bosch JM . Eosinophil cationic protein and immunoglobulin levels in bronchoalveolar lavage fluid obtained from patients with chronic eosinophilic pneumonia. Eur Respir J. 1996;9:2488‐2493. 8980958 10.1183/09031936.96.09122488

[46] van den Wall Bake AW , Black KP , Kulhavy R , Mestecky J , Jackson S . Transforming growth factor‐beta inhibits the production of IgG, IgM, and IgA in human lymphocyte cultures. Cell Immunol. 1992;144:417‐428. 1394452 10.1016/0008-8749(92)90256-o

[47] Hashiguchi M , Kashiwakura Y , Kanno Y , Kojima H , Kobata T . Tumor necrosis factor superfamily member (TNFSF) 13 (APRIL) and TNFSF13B (BAFF) downregulate homeostatic immunoglobulin production in the intestines. Cell Immunol. 2018;323:41‐48. 29100594 10.1016/j.cellimm.2017.10.009

[48] Jung Y , Wen T , Mingler MK , et al. IL‐1beta in eosinophil‐mediated small intestinal homeostasis and IgA production. Mucosal Immunol. 2015;8:930‐942. 25563499 10.1038/mi.2014.123 PMC4481137

[49] Ansel KM , Harris RB , Cyster JG . CXCL13 is required for B1 cell homing, natural antibody production, and body cavity immunity. Immunity. 2002;16:67‐76. 11825566 10.1016/s1074-7613(01)00257-6

[50] Harriman GR , Kunimoto DY , Elliott JF , Paetkau V , Strober W . The role of IL‐5 in IgA B cell differentiation. J Immunol. 1988;140:3033‐3039. 3258891

