# Supplementary material for: The relationship between airway immunoglobulin activity and eosinophils in COPD
Source: J Cell Mol Med. 2020 Dec 27;25(4):2203–12. doi: 10.1111/jcmm.16206 (PMC7882983; doi:10.1111/jcmm.16206)
Supplement: Supplementary file 1 — Supplementary Material [file JCMM-25-2203-s001.docx]

**ONLINE DATA SUPPLEMENT**

**The relationship between airway immunoglobulin activity and eosinophils in COPD**

Thomas Southworth, Andrew Higham, Umme Kolsum, Jian Li, Thomas Scott, Josiah Dungwa, Sriram Sridhar, Tuyet-Hang Pham, Paul Newbold & Dave Singh

**METHODOLOGY**

1. **Epithelial brush and blood gene expression**

Bronchial epithelial brushings were collected from the lower lobes and lysed in RLT buffer (Qiagen, Manchester, UK) and blood was collected into PaxGene RNA tubes (BD biosciences, Wokingham, UK). Total RNA was extracted using ZR RNA MicroPrep kit (Zymo Research, Orange, CA, USA) and RNA-seq libraries were prepared using TruSeq Stranded mRNA Prep kit (Illumina, San Diego, CA, USA) per manufacturers’ protocols. To confirm RNA quality, RIN scores were evaluated using the 2100 Bioanalyzer system (Agilent, Santa Clara, CA, USA), with an acceptable cut-off criteria of ≥7.0. Paired-end sequencing (75 base pairs per end) with sequencing depth at 80 million reads was performed on the HiSeq2000 platform (Illumina, San Diego, CA, USA) to generate FASTQ files. These were aligned to human genome (version HG19) using HiSAT2 (John Hopkin’s University, Baltimore, MA, USA) and SAMtools (Genome Research Limited, Cambridge, UK). Normalized read counts were generated per transcript using DESeq2 (1). Read counts were then transformed to log2 scale (after adding 1 to account for zero read counts) and comparisons between eosinophil^high^ and eosinophil^low^ groups were assessed for the immunoglobulin genes *igha1, igha2, ighm, jchain, ighg1, ighg2, ighg3, ighg4 and pIgR*, along with genes associated with plasma cell activity.

1. **Bronchial biopsy Immunohistochemistry**

To evaluate histological quality of biopsy tissue, all samples were stained with heamotoxylin and eosin prior to immunohistochemical analysis; samples were rejected if epithelial or subepithelial tissue was absent. Sections of formalin fixed, paraffin embedded bronchial biopsies, from the right and left lower lobes, were de-waxed in xylene and rehydrated in graded alcohols. Heat induced antigen retrieval was performed using citrate buffer pH6, for IgHG2; EDTA buffer pH8, for IgM; and Tris-EDTA Buffer pH9, for IgA2. Slides were cooled for 20 min and washed in TBS-tween (0.05%) buffer. For IgHG1, enzyme activated antigen retrieval was performed using pepsin for 10 min (Abcam, Cambridge, UK) and then washed in wash buffer. Slides were incubated in BLOXALL blocking solution (VectorLabs, Peterborough, UK) for 10 min to block endogenous peroxidases, washed in TBS-tween (0.05%) and incubated in 1.5% normal horse serum (VectorLabs) for 30 min at room temperature. For antigen detection, slides were incubated with primary antibodes; rabbit monoclonal anti-IgGA2 (Clone RM125, Abcam),rabbit monoclonal anti-IgHG1 (Abbexa, Cambridge, UK), mouse monoclonal anti-IgHG2 (Abbexa) and mouse monoclonal anti-IgM (Clone M260, Abcam) at dilutions of 1:400, 1:6400, 1:800 and 1:200 respectively, in universal antibody diluent (Abcam) for 2 hours at room temperature. The immune reaction was detected by incubating for 30 min at room temperature with a ready-to-use peroxidase-labelled secondary reagent (ImmPRESS™ anti-rabbit for IgGA2 and IgHG1 or ImmPRESS™ anti-mouse for IgGM and IgHG2, VectorLabs). The slides were then washed in TBS-tween (0.05%) and incubated in DAB chromogen (VectorLabs). Slides were counterstained in Mayer's haematoxylin and coverslipped in DPX.

Digital micrographs were obtained using a Nikon Eclipse 80i microscope (Nikon UK Ltd, Surrey, UK) equipped with a QI imaging digital camera and Image Pro Plus 6.0 software (Media Cybernetics, Marlow UK). The entirety of each bronchial biopsy was imaged. Using the count size feature in ImagePro Plus 6.0 software, threshold values were selected to detect immunoreactivity above background levels. ImagePro Plus 6.0 software used threshold values to quantify the area of immunoreactivity and the intensity of immunoreactivity within the epithelium or sub-epithelium. Alternatively, the number of immunoreactive cells per area of tissue was quantified. Analysis was conducted by one observer who was blinded to the eosinophil sub-group. Ten percent of samples were randomly selected for quality control by a second observer, where a coefficient of variation of <10% was deemed acceptable.

1. **In vitro B-cell treatment**

B-cells were extracted from the blood of a healthy non-smoking volunteer using a CD19 microbeads (Miltenyi Biotech, Bisley, UK) and cells were suspended in RPMI media. 1x10^6^ B-cells were treated with BAL fluid (25% final concentration) an incubated at 37^O^C, 5% CO_2_ for 72 hours. Total RNA was purified from cell lysates using RNeasy kits (Qiagen, Crawley, UK) according to manufacturer’s instructions. DNA contamination was prevented by on-column addition of DNase (Qiagen, Crawley, UK) according to manufacturer’s instructions. Reverse transcription- was performed on 50 ng of RNA using the Verso cDNAkit (Thermo Scientific). cDNA was reacted with ABsolute blue qPCR mix (Thermo Scientific) in 25 µl reactions containing premade ABI Taqman gene expression assays for *XBP1* (Primers and probes from Applied Biosystems, Warrington, UK; Hs00231936_m1). None template control showed there was no amplification. Thermal cycling was carried out on a Stratagene MX3005P (Agilent Technologies, West Lothian, UK). Relative expression levels were determined using the ΔΔCt method normalizing to the recommended B-cell endogenous control, ubiquitin C (Hs00824723_m1) (2) and to untreated, time matched, control cells.

1. **NTHI-specific immunoglobulin binding assay**

100μl of heat killed NTHI (5x10^6^/ml; NCTC strain 12699, Public Health England) was incubated with 100μl of BAL fluid (30min; 25^o^C; 900rpm), before being washed in 2% bovine serum albumin in phosphate buffered saline (Sigma-Aldrich, Poole, UK). Opsonised bacteria were stained with FITC labelled antibodies against human IgA or IgM for 20 min at 4°C before washing. For IgG1 and IgG2 detection, mouse anti-human IgG1 or IgG2 antibodies were incubated for 20 min at 4°C, washed twice then incubated with a goat-anti-mouse IgG FITC for 20 min at 4°C. Following two additional washes, pellets were suspended in PBS/2% BSA prior to acquisition on a FACS Canto II flow cytometer (BD Biosciences, Wokingham, UK. Bacteria only and bacteria + conjugate only were prepared as controls. Data is presented as the percentage of bacterial cells showing positive opsonisation.

**DISCUSSION OF IMMUNOGLOBULIN REGULATORS**

The epithelial gene expression of *NOS2* and lymphotoxins *LTA* and *LTB* were lower in the eosinophil^low^ compared with the eosinophil^high^ group, whilst there were no differences for other immunoregulatory mediators. Eosinophil depeletion in mice resulted in lower sIgA levels, associated with a reduction in eosinophil-dependent mediators of IgA production, including iNOS, LTα and LTβ (3). Reduced *NOS2* expression can decrease B-cell viability and reduce IgA^+^ plasma cell numbers (4). LTα attenuation reduces IgA levels in a chronic cigarette smoke exposure model and LTαβ signalling drives B-cell recruitment into lymphoid follicles (5). Greater tissue and blood eosinophil counts in COPD are associated with higher T2 cytokine expression (6). Therefore, the observed increases in expression of *NOS2* may be due to T2 cytokine production since IL-13 can increase *NOS2* expression. LTβ can also be induced in eosinophils following IL-5 exposure (7), which may be an alternative reason for higher levels of LTβ gene expression in eosinophil^high^ patients.

|  |  | Eosinophil^high^ | Eosinophil^low^ | P |
| --- | --- | --- | --- | --- |
| **Blood cell gene expression** | IgHA1 | 15.61 +/- 1.38 | 15.08 +/- 0.80 | 0.156 |
|  | IgHA2 | 13.63 +/- 1.29 | 13.01 +/- 0.84 | 0.091 |
|  | IgHM | 15.09 +/- 0.94 | 14.92 +/- 0.59 | 0.509 |
|  | IgHG1 | 14.03 +/- 1.79 | 13.76 +/- 0.67 | 0.229 |
|  | IgHG2 | 12.99 +/- 1.30 | 12.35 +/- 0.91 | 0.086 |
|  | IgHG3 | 13.42 +/- 1.54 | 12.93 +/- 0.75 | 0.218 |
|  | IgHG4 | 10.41 +/- 1.72 | 9.73 +/- 0.86 | 0.129 |
| **Serum protein** | IgA | 2.6 (1.8-22) | 2.4 (0.7-7.5) | 0.071 |
|  | IgM | 1.9 +/- 1.3 | 1.9 +/- 1.0 | 0.928 |

**Online Supplementary Table 1: Immunoglobulin gene and protein expression in blood.** Data presented as mean +/- standard deviation with comparisons between eosinophil^high^ and eosinophil^low^ by T-test, except for IgA protein, which is presented as median (range) and analysed by Mann-Whitney test.

**Online Supplementary Figure 1: Immunoglobulin levels in BAL of eosinophil^high^ and eosinophil^low^ COPD patients.** IgA (A) and IgM (B) levels in BAL were measured by Myriad RBM assay, while secretory IgA (C), IgG1 (D) and IgG2 (E) were assessed by ELISA. Comparison between eosinophil high and low patients was by t-test for IgA, IgM and secretory IgA, and Mann-Whitney for IgG1 and IgG2: *p<0.05; **p<0.01. Bars illustrated mean value for IgA, IgM and secretory IgA, and median for IgG1 and IgG2.

**
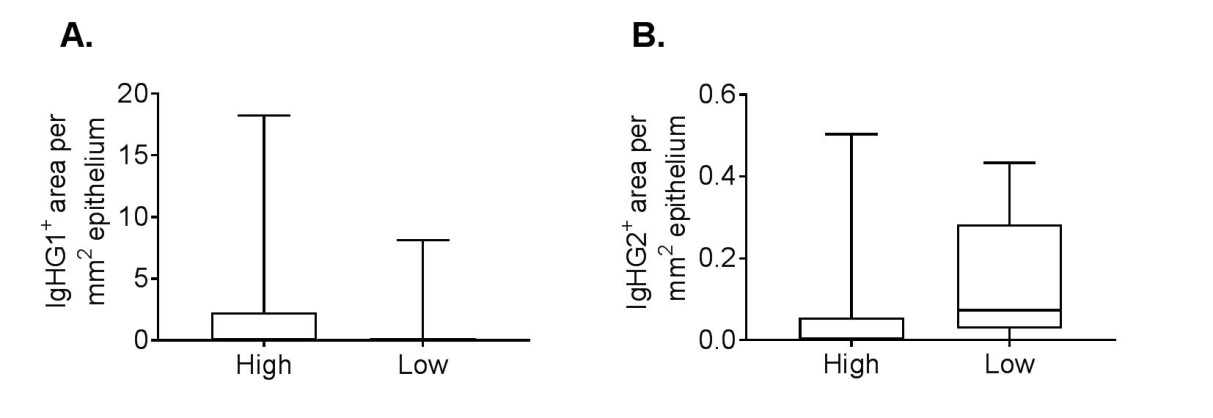
**

**Online Supplementary Figure 2: Analysis of epithelial IgG1 and IgG2 expression in bronchial biopsies from** **eosinophil^high^ and eosinophil^low^ patients.** Positive expression of epithelial IgG1 (A) and IgG2 (B) were calculated following immunohistochemical staining. Comparisons between eosinophil^high^ and eosinophil^low^ were by Mann-Whitney test: *p<0.05. Box and whisker plots represent median, inter-quartile range and range.

**Online Data Supplement References**

1. Love MI, Huber W, Anders S. Moderated estimation of fold change and dispersion for RNA-seq data with DESeq2. *Genome Biol* 2014; 15: 550.

2. Oturai DB, Sondergaard HB, Bornsen L, Sellebjerg F, Christensen JR. Identification of Suitable Reference Genes for Peripheral Blood Mononuclear Cell Subset Studies in Multiple Sclerosis. *Scand J Immunol* 2016; 83: 72-80.

3. Jung Y, Wen T, Mingler MK, Caldwell JM, Wang YH, Chaplin DD, Lee EH, Jang MH, Woo SY, Seoh JY, Miyasaka M, Rothenberg ME. IL-1beta in eosinophil-mediated small intestinal homeostasis and IgA production. *Mucosal Immunol* 2015; 8: 930-942.

4. Gommerman JL, Rojas OL, Fritz JH. Re-thinking the functions of IgA(+) plasma cells. *Gut Microbes* 2014; 5: 652-662.

5. Demoor T, Bracke KR, Maes T, Vandooren B, Elewaut D, Pilette C, Joos GF, Brusselle GG. Role of lymphotoxin-alpha in cigarette smoke-induced inflammation and lymphoid neogenesis. *Eur Respir J* 2009; 34: 405-416.

6. Christenson SA, Steiling K, van den Berge M, Hijazi K, Hiemstra PS, Postma DS, Lenburg ME, Spira A, Woodruff PG. Asthma-COPD overlap. Clinical relevance of genomic signatures of type 2 inflammation in chronic obstructive pulmonary disease. *Am J Respir Crit Care Med* 2015; 191: 758-766.

7. Bates ME, Liu LY, Esnault S, Stout BA, Fonkem E, Kung V, Sedgwick JB, Kelly EA, Bates DM, Malter JS, Busse WW, Bertics PJ. Expression of interleukin-5- and granulocyte macrophage-colony-stimulating factor-responsive genes in blood and airway eosinophils. *Am J Respir Cell Mol Biol* 2004; 30: 736-743.
